# Indirect Acquisition of Pain-Related Fear: An Experimental Study of Observational Learning Using Coloured Cold Metal Bars

**DOI:** 10.1371/journal.pone.0117236

**Published:** 2015-03-25

**Authors:** Kim Helsen, Johan W. S. Vlaeyen, Liesbet Goubert

**Affiliations:** 1 Research Group on Health Psychology, KU Leuven - University of Leuven, Tiensestraat 102, Leuven, Belgium; 2 Department of Experimental-Clinical and Health Psychology, Ghent University, Henri Dunantlaan 2, Ghent, Belgium; 3 Department of Clinical Psychological Science, Maastricht University, Universiteitssingel 40, Maastricht, Netherlands; University of Akron, UNITED STATES

## Abstract

**Background:**

Previous research has demonstrated that pain-related fear can be acquired through observation of another’s pain behaviour during an encounter with a painful stimulus. The results of two experimental studies were presented, each with a different pain stimulus, of which the aim was to investigate the effect of observational learning on pain expectancies, avoidance behaviour, and physiological responding. Additionally, the study investigated whether certain individuals are at heightened risk to develop pain-related fear through observation. Finally, changes in pain-related fear and pain intensity after exposure to the feared stimulus were examined.

**Methods:**

During observational acquisition, healthy female participants watched a video showing coloured cold metal bars being placed against the neck of several models. In a differential fear conditioning paradigm, one colour was paired with painful facial expressions, and another colour was paired with neutral facial expressions of the video models. During exposure, both metal bars with equal temperatures (-25° or +8° Celsius) were placed repeatedly against participants’ own neck.

**Results:**

Results showed that pain-related beliefs can be acquired by observing pain in others, but do not necessarily cause behavioural changes. Additionally, dispositional empathy might play a role in the acquisition of these beliefs. Furthermore, skin conductance responses were higher when exposed to the pain-associated bar, but only in one of two experiments. Differential pain-related beliefs rapidly disappeared after first-hand exposure to the stimuli.

**Conclusions:**

This study enhances our understanding of pain-related fear acquisition and subsequent exposure to the feared stimulus, providing leads for pain prevention and management strategies.

## Introduction

Chronic pain is one of the major health problems in Western societies, with a prevalence of 19% [1,2,3]. Not only does chronic pain account for enormous health care costs and lost working productivity, it also results in substantial quality of life reduction [1,4]. An important predictor in the development as well as the continuation of pain problems is pain-related fear [5,6]. This fear instigates catastrophic ruminations about pain and avoidance behaviour which interfere with cognitive, physical and social functioning [7,8]. Despite its demonstrated importance, yet little is known about how pain-related fear is acquired in the first place.

In accordance with Rachman’s three pathways theory of fear acquisition, pain-related fear is expected to be acquired through direct experience [9,10], verbal instruction [11], and observation [12]. This latter type of learning was described by Bandura (p. 49) [13] as ‘*changes in cognitive skills or patterns of behaviour that are a consequence of observing others’ performances*’. Behavioural responses may reflect automatic or reflexive processes, neuronal activity, or behaviours following deliberate control [14].

Observational learning is important in shaping an individual’s pain response and experience. Previous studies have mainly focused on the influence of modelling on pain intensity, pain threshold, or pain tolerance. For example, Craig and Weiss [15] examined the impact of pain tolerant and intolerant models on students’ verbal pain reports induced by electrical pain stimulation. A significant impact was found on both pain expressions and willingness to accept pain stimuli of increased intensity. Observing tolerant models also led to a reduction in subjective distress [16]. However, these studies about observational learning and pain did not provide information about the development of *fear* of pain.

Recently, researchers have shown increased interest in the observational learning pathway to pain-related fear [17], although empirical evidence is relatively scarce. Olsson et al. [18] systematically investigated different pathways leading to pain-related fear. Comparisons between these learning types (operationalized by changes in skin conductance) revealed that observational and verbal fear learning can be as effective as aversive learning through first-hand experience. In a previous study using coloured cold pressor tasks (CPT) [19], evidence was found for the acquisition of fearful expectancies through observation. Participants watched models displaying painful facial expressions during immersion of the hand in a CPT with one colour (CS+), and neutral expressions during a CPT with another colour (CS-). Despite differential pain expectancies, no differences in avoidance behaviour were observed. After watching the video, participants were requested to consecutively immerse their hand in each CPT. Notwithstanding equal temperatures, more pain-related fear was expressed when exposed to the CS+ CPT. In other words, fear of pain did not extinguish completely after one exposure to the feared stimulus, although the difference in reported fear was much smaller after the immersion compared to immediately after watching the observation video.

The main objective of the current study was to conceptually replicate and extend earlier findings. The first aim was to find evidence for observational acquisition of pain-related fear. Secondly, the study investigated whether observationally acquired pain-related fear gradually extinguishes after direct, repeated contact with the feared stimulus. Cold metal bars were chosen as stimuli, because they can be repeatedly presented for a short period of time. Similar stimuli have been used successfully in a study by Arntz and Claassen [20], who investigated whether painfulness of ambiguous stimuli depends on the meaning attached to them. If pain-related fear can be extinguished after direct contact to the feared stimulus, exposure therapy, during which pain patients perform feared movements despite pain, might be a promising behavioural treatment in clinical practice. Next to measures of fear, pain expectancy and avoidance behaviour, psychophysiological responding was examined with respect to these first two aims. Finally, several observers’ characteristics were expected to influence observational learning. First, pain catastrophising, which can be defined as a negative cognitive-affective response to anticipated or actual pain [21], is suggested to influence pain experiences and disability, putatively mediated by appraisals, such as pain-related fear beliefs [22]. Additionally, catastrophising has been found to enhance fear processing [23]. Second, individuals with higher trait fear of pain may experience an aversive stimulus as more threatening, resulting in a stronger conditioned response. In prior research, trait fear of pain was associated with increased pain intensity ratings [24]. Third, trait negative affectivity (NA) is a general dimension of subjective distress that subsumes a diversity of aversive mood states, including fear and anxiety [25]. Increasing evidence has been found for NA as a moderator in the acquisition of pain-related fear, probably through attentional processes [8,26]. Evidence for the importance of these three characteristics in an individual’s pain experience can be found in studies concerning the Fear-Avoidance model of chronic pain [27,28]. Fourth, dispositional empathy has been suggested to be a condition for observational learning to occur [29,30]. Examining these characteristics is essential in the identification of individuals at risk of developing pain problems.

A differential fear conditioning paradigm was employed, during which participants watched video models exposed to one of two coloured metal bars (briefly placed in their neck), displaying either a painful (CS+ colour) or a neutral (CS- colour) facial expression (observation phase). Afterwards, participants were directly and repeatedly exposed to both metal bars, which had equal temperatures (exposure phase).

Regarding the first aim, during the observation phase, participants were expected to experience more pain-related fear regarding the CS+, and to expect contact with this bar to be more painful than the CS- bar, eliciting stronger avoidance behaviour. With respect to the second aim, during the exposure phase, differences in pain-related fear, skin conductance responses and pain intensity between the CS+ and the CS- bar were hypothesized to diminish gradually due to equal temperature of the stimuli. Concerning the final aim, higher levels of pain catastrophising, trait fear of pain, negative affectivity, and dispositional empathy in the observer were expected to facilitate observational learning.

In the current study, two experiments were conducted with different bar temperatures (-25°C and 8°C) to eliminate the possibility that the absence of differential responding was merely due to the temperature of the stimuli.

## Experiment 1: Method

### Participants

Participants were healthy female undergraduate students (N = 49), who received either a course credit or eight Euros for their participation in the study. Exclusion criteria were the experience of chronic pain and colour blindness. All participants were of Western European descent, with a mean age of 20.47 years (*SD* = 3.76, range 15–42). They were told that the study investigated responses to stimuli of different temperatures.

### Ethics statement

**Fig 1:**
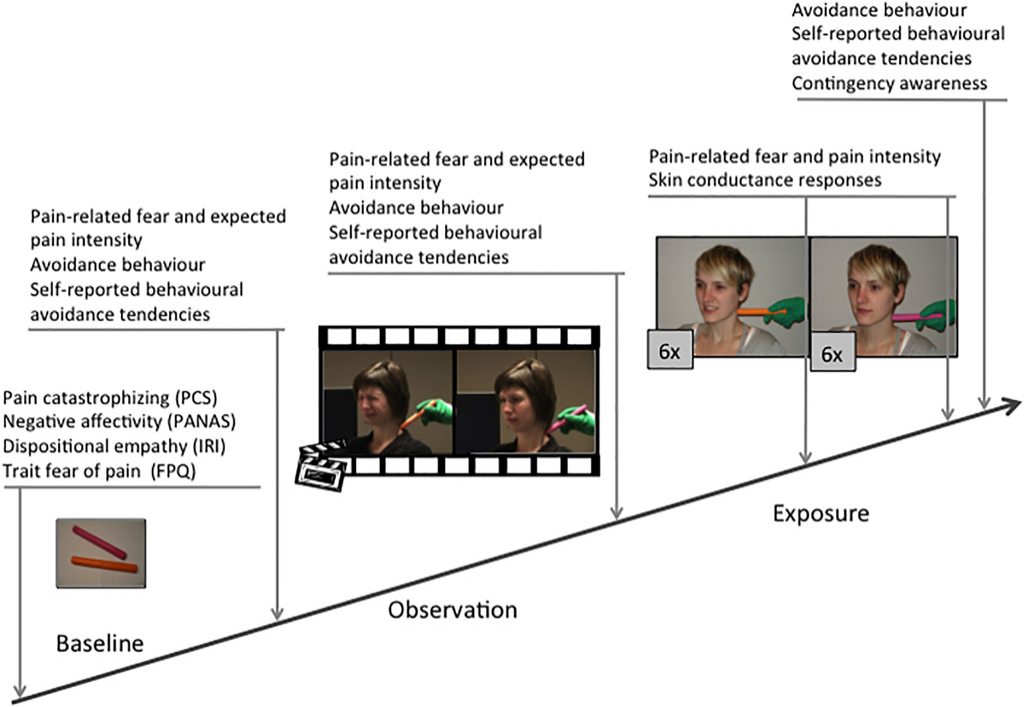
Graphical overview of the experimental procedure, with the measurements during the baseline, observation, and exposure phase. During the observation phase, one colour was associated with painful facial expressions of the video models (left), while the other colour was paired with neutral expressions (right).

Ethical approval for Experiment 1 was obtained from the Ethics Committee of the Faculty of Psychology and Educational Sciences of the University of Leuven, Belgium (Reg. nr. S52888). Participants signed the informed consent form. The individuals shown in Fig. 1 are researchers who were either a video model in the study (Observation) or pretended to be a participant (Exposure), and provided written informed consent to publish these pictures.

### Materials

#### Observation Video

An observation video clip was developed for this study, showing four female human models being exposed to coloured metal bars (conditioned stimuli, CS) in a similar way as in a previous study by Arntz and Claassen [20]. Models were females with a mean age of 24.25 years (*SD* = 2.22, range 22–27) who were requested to mimic painful facial expressions that served as the unconditioned stimuli (US). Considering that participants were also young females, we expected identification between models and participants. One coloured bar was always associated with painful facial expressions (CS+), the other with neutral facial expressions (CS-). Each model was shown three times, which resulted in a 12-trial video clip. The CS+ and CS- metal bars were presented six times each, with a maximum of two consecutive trials of the same type. The colour of the CS+ was counterbalanced: Half of the participants watched a video with an orange metal bar associated with the painful facial expressions; the other half watched a video in which a pink metal bar was associated with the pain expressions. Total duration of the video was about 5 minutes, with trial time varying from 15 to 26 seconds.

#### Conditioned Stimuli

The metal bars (aluminium, length 17.0 cm, diameter 2.0 cm), which were placed against participants’ neck, were coloured with a spray (Motip Dupli, orange, 466663; pink, 470998) and cooled down in a freezer to approximately -25°C. A previous study [20] showed that an exposure time of one second at this temperature creates an ambiguous stimulus. The same bars were used in the observation video. During the observation video as well as during the actual exposure to participants’ neck, not more than two consecutive trials of the same coloured bar were presented.

### Measures

#### Contingency Awareness

Participants’ awareness of the contingency between the colour of the metal bars and the facial expressions of the video models (painful vs. neutral) was measured with a categorisation task. At the end of the experiment, participants were shown one black-and-white picture of each video extract, with clearly visible facial expressions of the models being touched by the bars. They were requested to categorise these pictures into two piles: one pile was associated with the pink, and the other with the orange metal bar. Afterwards, they were asked which criterion they had used to categorise the pictures.

#### Self-reports

Numerical rating scales (NRS) regarding properties of being in contact with the metal bars (pain-related fear and pain intensity) were presented. NRS are a common, practical and valid way to investigate pain-related outcomes [31,32,33]. The scales ranged from 0 (*not fearful/painful at all*) to 10 (*very fearful/painful*). During the baseline and observation phase, these scales referred to participants’ expectations, while during the exposure phase, the scales inquired about their actual experiences. At the end of each phase, self-reported behavioural avoidance tendencies (BAT) were measured, with participants rating on a NRS, ranging from 0 (*not at all*) to 10 (*very much*), their willingness to touch each metal bar.

#### Avoidance Behaviour: The Approach-Avoidance Task (AAT)

The approach-avoidance task, used to assess approach and avoidance tendencies regarding particular stimuli, is a categorisation task based on the compatibility principle. This means that, although the content of the presented stimuli is irrelevant for the instruction of the task, participants’ reaction time is affected by the compatibility between the response and the valence of the stimuli [34]. The task used in the current experiment was adapted from Rinck and Becker [35], and compatibility scores were used as an indirect behavioural measure for pain-related fear.

For this task, a joystick (Logitech, type Attach 3TM) was positioned between the participant and a computer screen. Pictures of six stimuli, including the two coloured metal bars used in the experiment, were presented on the screen, either vertically or horizontally oriented. The pictures of the four additional stimuli (filler stimuli) represented neutral objects that one could easily categorise as being horizontally or vertically oriented (an apple corer, a pepper mill, a pencil, and a spoon). In order to create a zooming effect to simulate the approach or avoidance of a particular stimulus, as was done previously by Rinck and Becker [35], every picture was available in seven pixel sizes (99x132, 165x220, 225x300, 300x400, 360x480, 420x560, 510x680), and every picture size corresponded to one of seven imaginary regions on the computer screen (10–110, 110–210, 210–310, 310–458, 458–558, 558–658, 658–758 pixels height, respectively). There were two imaginary end regions: one at the top (0–10 pixels), and one at the bottom (758–768 pixels) of the screen. Each trial was initiated by the participant pushing the start button of the joystick, which was then followed by the display of the medium-sized picture. Picture size changed whenever the hidden cursor of the joystick entered a different region. For instance, when pushing the joystick away, the cursor entered the 310–210 pixel region, which resulted in presentation of the smaller 225x300 pixel size picture. When pushing the joystick even further, picture size further decreased, which seemed to enlarge the distance between the participant and the stimulus. When entering the end region, the picture disappeared, irrespective of the response accuracy. Similarly, when participants pulled the joystick towards themselves, the picture size gradually increased, which gave the impression of an approaching stimulus. Movements to the left or right had no effect.

Participants were instructed to pull the joystick towards themselves whenever they saw a picture of a vertically oriented object on the computer screen, and to push the joystick away when a horizontally oriented object was shown, or vice versa (counterbalanced). They were requested to do this as quickly and accurately as possible. The task, which comprised 88 trials, was administered in each of the three phases (baseline, observation, and exposure). The two pictures showing a metal bar were presented 10 times in both horizontal and vertical orientation. The four filler stimuli were presented six times in each orientation. Pictures were presented randomly with the restrictions that the first two pictures never displayed a metal bar, and that consecutive trials did not show the same picture. During the baseline phase, a practice phase consisting of 12 trails preceded the actual AAT: Every stimulus was randomly presented in both orientations. Only during these practice trials, participants received feedback about the accuracy of their answers.

#### Physiological arousal: Skin Conductance Responses

During the exposure phase, skin conductance responses (SCR) to the pictures of the metal bars measured the level of participant’s arousal while they were anticipating the actual exposure to each stimulus, while SCR to the presentations of the metal bars were measured to determine the level of participant’s arousal during the actual exposure.

Electrodermal activity was measured using the Coulbourn skin conductance coupler (V71–23). Two standard Ag/AgCl electrodes (diameter 0.8 cm), with an inter-electrode distance of 2.0 cm, were filled with KY gel (Johnson & Johnson), and placed on the hypothenar eminence of the non-dominant hand, which was scrubbed and cleaned with tap water before the start of the experiment. The skin conductance coupler maintained a constant 0.5 V across the electrodes. The analogue signal was converted with a 12-bit AD-transducer and digitised at 10 Hz. Skin conductance was recorded using Affect 4.0 software [36] and treated offline with Psychophysiological Analysis software (PSPHA) [37].

#### Observers’ Characteristics

The Pain Catastrophizing Scale (PCS) [38,39] is a 13-item self-report measure used in both clinical and non-clinical populations to assess catastrophic thinking about pain. Participants were asked to indicate on a 5-point scale (0 = not at all; 4 = always) the degree to which they experienced negative thoughts and feelings during painful situations. Although three subscales can be distinguished (rumination, magnification, and helplessness), only the total score was of interest in the current study. High internal consistency of this sum score was found (Cronbach’s alpha = .87), which is comparable to the reliability found in previous studies (Cronbach’s alpha = .85 – .91) [40,41].

Trait fear of pain was measured by means of the Fear of Pain Questionnaire (FPQ-III) [42,43], which includes 31 descriptions of specific painful situations. Participants were requested to rate the degree of fear they anticipated to experience in each situation (A = no fear at all; E = extreme fear). Reliability of the total score was very good (Cronbach’s alpha = .90). Previous studies have shown good reliability and validity of the FPQ-III in both clinical and non-clinical populations [42,44].

In order to assess trait negative affectivity, the Trait version of the Positive And Negative Affect Schedule (PANAS) [25,45] was administered. Participants reported the degree by which they experienced 20 different emotions in daily life (very little; very often). Half of the adverbs described positive emotions, the other half negative emotions. In the current study, only the Negative Affectivity subscale (PANAS-NA) was utilised, containing the sum score of the 10 negative adverbs. Internal consistency of this subscale was good (Cronbach’s alpha = .82), which is comparable to previous research (Cronbach’s alpha = .83–.87) [25,45].

The Interpersonal Reactivity Index (IRI) [46–48] is a self-report measure to assess dispositional empathy, consisting of 28 reflecting thoughts and feelings one can experience in interpersonal contexts. Participants were asked to indicate to what extent the statements described them (A = does not describe me well; E = describes me very well). The IRI encompasses four subscales: Perspective Taking (PT; i.e., the tendency to adopt another’s psychological point of view), Fantasy (FS; i.e., the tendency to identify strongly with the feelings and actions of fictitious characters), Empathic Concern (EC; i.e., the tendency to experience feelings of warmth, sympathy, and concern for unfortunate others), and Personal Distress (PD; i.e., the tendency to experience feelings of discomfort and concern when witnessing others’ distress) [46]. Cronbach’s alphas for the separate subscales were .77, .81, .70, .64 (Experiment 1), and .78, .90, .76, .81 (Experiment 2), respectively. Previous studies using the Dutch IRI have also found satisfactory internal consistency (Cronbach’s alphas = .73, .83, .73, .77, respectively) [48].

### Procedure

After being informed about the course of the experiment, participants signed the informed consent form. Prior to the start of the experiment, the questionnaires (PCS, FPQ, IRI, and PANAS) were completed. Participants were then asked to scrub the palm of the non-dominant hand and to rinse it with tap water before the electrodes were attached to the hypothenar eminence.

As shown in Fig. 1, the experiment consisted of three phases: (1) baseline, (2) observation, and (3) exposure. During the *baseline phase*, participants were requested to report their pain-related fear and expected pain intensity concerning being in contact with the metal bars. Next, the AAT was administered. At the end of the baseline phase, participants rated their willingness to touch both bars (self-reported behavioural avoidance tendency). During the *observation phase*, participants watched the models in the video clip showing either painful or neutral facial expressions when exposed to the CS+ and CS- metal bar respectively. After rating the self-reported expectancies regarding pain-related fear and pain intensity, the AAT was performed, and self-reported avoidance tendencies were measured. During the *exposure phase*, both metal bars were placed repeatedly against participants’ neck. At the beginning of each trial, a picture showing the upcoming bar appeared on the computer screen for five seconds. 30 seconds later, the participant was exposed to the bar for one second. Immediately after each exposure, participants were asked to report the degree of pain-related fear and pain intensity they had experienced. Skin conductance was recorded throughout this phase. Once the 12 exposure trials were completed, participants completed the AAT, and reported avoidance tendencies regarding both stimuli. At the end of the experiment, contingency awareness was checked, and participants were debriefed about the broader context and purpose of the study.

### Data Preparation and Statistical Analyses

Concerning the *AAT*, median reaction times per stimulus type (CS+ vs. CS-), per response direction (pulling vs. pushing the joystick), were determined for each participant, excluding reaction times of incorrect responses. Subsequently, median scores in the pull condition were subtracted from medians in the push condition for each stimulus type separately to compute compatibility scores. As a result, the relative strength of approach and avoidance regarding the three stimulus types was measured, with positive scores representing stronger approach tendency, and negative scores representing stronger avoidance. *SCR data* were determined with respect to both the pictures of the metal bars and the actual presentations of the bars during the exposure phase. Concerning the *SCR to the pictures*, the mean value in the 2-second window before presentation of the picture was compared to the maximum value of the 8-second window after presentation of either picture. Concerning the *SCR to the presentations of the bars*, the mean value in the 2-second window before presentation of each bar was compared to the maximum value of the 4-second window after presentation of either bar. The time window after presentation of each bar was restricted to four seconds, because numerical rating scales were presented after five seconds. Hence, extending this time window would lead to interference of the SCR with reactions to the presentation of novel stimuli (the scales), or movements associated with answering these scales. A logarithmic transformation (Log10(SCR+1)) was performed on the SCR data before statistical testing to reduce skewness.

Mixed model statistical analyses were conducted with stimulus type (CS+ versus CS-) and time (baseline, observation, and exposure) as within-subject factors. For each dependent variable, two models were compared: (1) stimulus type, time and stimulus type x time as fixed effects, and intercept as a random effect, (2) stimulus type, time and stimulus type x time as fixed effects, and intercept and time as random effects. The model with the significantly lowest (full) maximum likelihood produces the best fit. For most dependent variables, the first model (with the random intercept and fixed slope) yielded the best fit. Hence, in order to preserve consistency, all analyses were conducted using this model. The same model was also used in moderation analyses, entering centred PCS, FPQ, IRI or PANAS-NA scores as covariates. Significant statistical interactions between stimulus type and questionnaire scores denoted moderation effects. Regression analyses were conducted separately for each stimulus type to explore the direction of these effects according to the procedure described by Baron and Kenny [49]. Differential effects (CS+ vs. CS-) for individuals scoring higher or lower on the moderator variable were investigated by centring the covariates around the -1 *SD* (lower moderator scores) or +1 *SD* value (higher moderator scores). Effect sizes *r* were computed using the formula provided by Kenny et al. [50], with *r* = .10 indicating a small effect, *r* = .30 a medium effect, and *r* = .50 a large effect [51].

*Self-reported acquisition* of pain-related fear and expected pain intensity were investigated analysing both the baseline and observation phase. Successful acquisition would be reflected by a significant interaction between stimulus type and time. For the six *self-report measurements during exposure*, baseline scores, and the interaction between baseline score and stimulus type were included as additional factors. Baseline effects not reaching statistical significance were deleted from moderation analyses for the corresponding dependent variable. Throughout the exposure phase, *psychophysiological responses* were investigated comparing SCR to the pictures and actual presentations of the six CS+ and six CS- trials. With respect to the analyses of the *self-reported behavioural avoidance tendencies* (BAT), and *the AAT compatibility scores*, all three phases were included together in the analyses. 3-way interactions between stimulus type, time, and scores on the questionnaires were included to examine whether the effects of the observers’ characteristics on the relationship between stimulus type and the BAT and compatibility scores varied across time. If the 3-way interaction was significant, a distinction was made between acquisition (baseline x observation phase) and exposure to explore when the effect of the moderator was strongest.

All analyses were conducted with an alpha ≤ 0.05, using SPSS 19.0. Bonferroni corrections were implemented in all pairwise comparisons. The use of mixed model analyses may result in the report of fractionated denominator degrees of freedom, which are obtained by a Satterthwaite approximation [52].

## Experiment 1: Results

### Sample Characteristics

Descriptive statistics, internal consistency, and Pearson inter-correlations regarding the questionnaire total scores and subscales are summarised in Table 1. Mean scores were comparable to what has been reported in previous research [38,43,45,48].

**Table 1:** Means (M), Standard Deviations (SD), Cronbach’s Alpha, and Pearson Intercorrelations of the Questionnaires.

	Experiment 1			Experiment 2									
	*M*	*SD*	Cronbach’s alpha	*M*	*SD*	Cronbach’s alpha	1	2	3	4	5	6	7
1 PCS	15.45	7.40	.87	14.12	6.50	.83		.30*	-.03	-.06	-.11	.14	.34*
2 FPQ	77.21	13.90	.90	74.51	14.23	.90	.39**		-.06	-.10	-.12	.24**	.39**
3 IRI PT	16.84	4.60	.77	16.37	4.27	.78	-.25	-.33*		.10	.37**	.41**	.06
4 IRI FS	18.98	4.96	.81	18.72	5.84	.90	.14	.25	.05		.40**	.21	.13
5 IRI EC	18.98	3.94	.70	19.19	4.30	.76	.06	.27	.40**	.30*		.26	.07
6 IRI PD	14.47	3.66	.64	13.23	4.62	.81	.01	.47**	.01	.19	.48**		.48**
7 PANAS NA	20.12	5.32	.82	21.05	6.94	.87	.26	.39**	-.26	.36*	.13	.27	

*Note*. The intercorrelation values above the diagonal represent the scores of Experiment 1, whereas the values below the diagonal show intercorrelations of Experiment 2. PCS = Pain Catastrophising Scale, FPQ = Fear of Pain Questionnaire, IRI = Interpersonal Reactivity Index, PT = Perspective Taking, FS = Fantasy, EC = Empathic Concern, PD = Personal distress, and PANAS-NA = Positive And Negative Affect Schedule—Negative Affectivity subscale.

* p < 0.05,

** *p* < 0.01.

### Contingency Awareness

All participants reported awareness of the contingency between the colour of the metal bars and the facial expressions of the video models (painful versus neutral). Categorisation data was available for 45 participants (91.8%), and revealed that 62.2% of these participants correctly categorised all six CS+ pictures as painful, 28.9% made one error, 6.7% made two errors, and 2.2% made more than two errors.

### Self-reported Pain-related Fear and Pain Intensity

#### Baseline and Observation Phase

**Fig 2:**
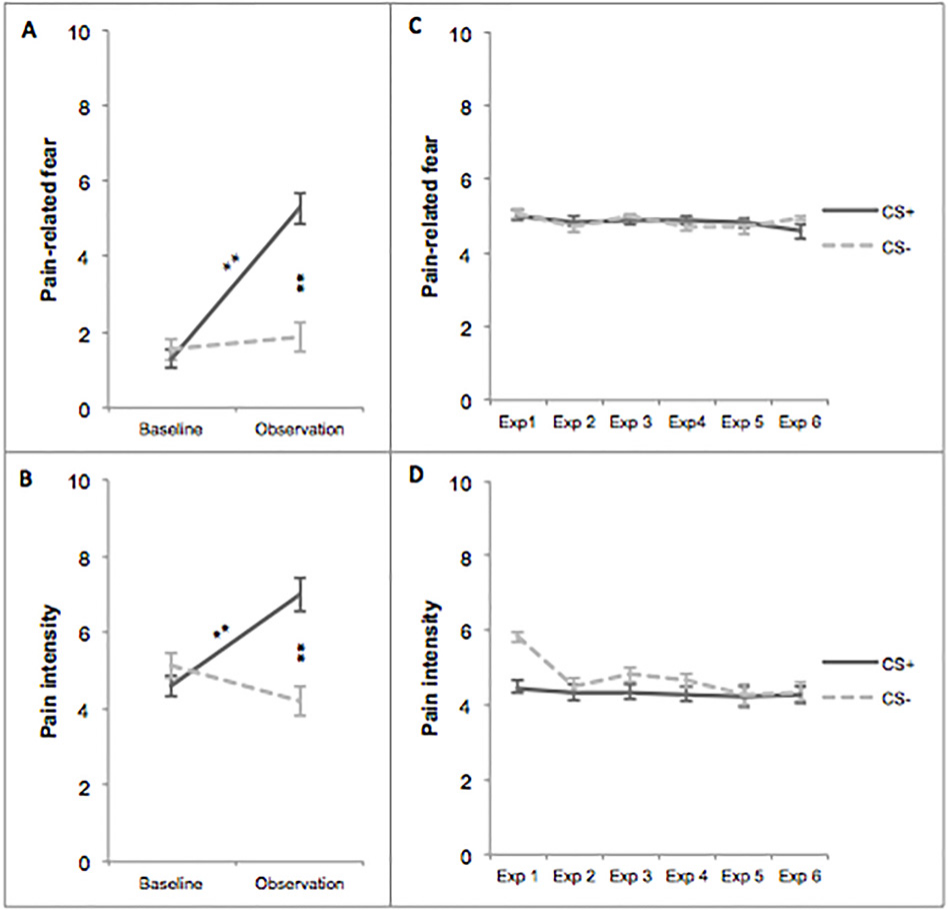
Self-reports: Pain-related fear and pain intensity in Experiment 1. Error bars represent standard errors. Exp = Exposure; * *p* < .05; ** *p* < .001.

As can be seen in Fig. 2A, concerning **pain-related fear**, main effects of stimulus type and time were found, *F*(1;145.54) = 23.83, *p* < .001, effect size *r* = .53; *F*(1;147.02) = 43.83, *p* < .001, *r* = .05, respectively. In addition, a significant interaction was found between stimulus type and time, *F*(1;145.54) = 32.46, *p* < .001, *r* = .43. Participants reported no difference in fear between both stimulus types during baseline, *F*(1;49) = 1.39, *p* = .24. During the observation phase, however, more fear was reported concerning the CS+ bar compared to the CS- bar, *F*(1;96) = 37.10, *p* < .001, *r* = .53. This difference was due to an increase in fear regarding the CS+, *F*(1;97) = 71.50, *p* < .001, *r* = .65, as no difference was found between the two phases with respect to the CS-, *F*(1;48.75) = 0.61, *p* = .44.

Furthermore, Fig. 2B illustrates the main effects of stimulus type and time that were found on expected **pain intensity**, *F*(1;145.60) = 29.32, *p* < .001, *r* = .54; *F*(1;147.09) = 33.00, *p* < .001, *r* = .02, respectively. In addition, a significant interaction was found between stimulus type and time, *F*(1;145.60) = 37.37, *p* < .001, *r* = .44. During the baseline, participants reported no difference in expected pain intensity between both stimulus types, *F*(1; 49) = 1.62, *p* = .21, whereas during the observation phase, contact with the CS+ bar was expected to be more painful than the CS- bar, *F*(1;96) = 48.22, *p* < .001, *r* = .58. This differential effect was caused by an increase in pain intensity expectancy regarding the CS+, *F*(1;49.16) = 74.27, *p* < .001, *r* = .72, as no difference between baseline and observation was found for the CS- bar, *F*(1;48.82) = 0.09, *p* = .76.

#### Influence of Observers’ Characteristics during the Baseline and Observation Phase

Putative moderating effects of pain catastrophising, trait fear of pain, dispositional empathy (PT, FS, EC, and PD), and negative affectivity in the observer were investigated. Only statistically significant effects are reported and explained. **Perspective taking** (PT) moderated the relationship between stimulus type and respectively pain-related fear, *F*(1; 145.54) = 8.67, *p* < .01, *r* = .27, and expected pain intensity, *F*(1; 145.59) = 10.18, *p* < .01, *r* = .29. Participants with lower PT scores reported more fear, and expected greater pain intensity regarding the CS+ compared to the CS-, *F*(1; 46) = 11.44, *p* < .001; *F*(1; 46) = 14.99, *p* < .001, respectively, whereas for individuals with higher PT, no difference between CS+ and CS- was observed, *F*(1; 46) = 0.06, *p* = .81; *F*(1; 46) = 0.11, *p* = .75, respectively. With respect to the CS+, no difference on pain-related fear (β = -.09, 39) or pain intensity (β = -.11, 30) was found between participants scoring higher or lower on PT. With respect to the CS-, however, participants scoring higher on PT were more afraid of being touched by the CS- bar (β = .26, *p* = .01), and expected it to be more intense (β = .28, *p* < .01) compared to participants scoring lower on PT.

#### Exposure Phase

With respect to **pain-related fear** and **pain intensity**, no main effects of stimulus type (pain-related fear: *F*(1;570.07) = 0.21, *p* = .65; pain intensity: *F*(1;561.31) = 1.59, *p* = .21) and time (pain-related fear: *F*(5;527.68) = 1.55, *p* = .17; pain intensity: *F*(5;527.58) = 1.52, *p* = .18), nor an interaction between these two variables was found (pain-related fear: *F*(5;527.68) = 1.23, *p* = .30; pain intensity: *F*(5;527.58) = 0.89, *p* = .48). This was illustrated in Fig. 2 (i.e., 2C and 2D).

#### Influence of Observers’ Characteristics during the Exposure Phase

The same putative moderating influences were examined during the exposure phase. Only statistically significant effects are reported. **Personal distress** (PD) moderated the relationship between stimulus type and respectively pain-related fear and pain intensity, *F*(1;528) = 7.48, *p* = .01, *r* = .26; *F*(1;528) = 5.57, *p* = .02, *r* = .23. Participants with lower PD reported more pain-related fear with regard to the CS+ compared to the CS-, *F*(1; 46) = 4.58, *p* = .04, whereas participants with higher PD reported more pain-related fear with respect to the CS- relative to the CS+ *F*(1; 46) = 6.79, *p* = .01. Regarding pain intensity, no differential effects were found for participants scoring higher (*F*(1; 46) = 2.89, *p* = .10) or lower (*F*(1; 46) = 0.25, *p* = .62) on PD. Regarding the CS+, no difference on pain-related fear was found between lower and higher PD (β = -.05, *p* = .43). However, participants with higher PD scores reported more pain after contact with the CS+ bar compared to individuals scoring lower (β = .15, *p* = .01). Regarding the CS- bar, participants with lower PD reported less fear during the exposure phase compared to participants with higher PD (β = .18, *p* < .01). No difference was found between participants scoring lower or higher on PD with respect to CS- associated pain intensity (β = -.03, *p* = .60). Furthermore, **trait fear of pain** was found to moderate the relationship between stimulus type and pain intensity, *F*(1;517) = 5.02, *p* = .03, *r* = .22. Participants with lower trait fear of pain perceived more intense pain regarding the CS- compared to the CS+ metal bar, *F*(1; 45) = 10.34, *p* < .01. For participants with higher trait fear of pain, no differential effects were found, *F*(1; 45) = 0.01, *p* = .94. Regarding the CS+, participants with lower trait fear of pain reported less pain intensity than participants with higher FPQ scores (β = .17, *p* < .01). No evidence for a difference on pain intensity between lower and higher scorers was found with respect to the CS- bar (β = .001, *p* = .98). Finally, **negative affectivity** (NA) was found to moderate the relationship between stimulus type and pain-related fear, *F*(1;528) = 4.70, *p* = .03, *r* = .21. No difference in pain-related fear between the CS+ and CS- bar was found for participants with higher or lower NA, *F*(1; 46) = 2.73, *p* = .11; *F*(1; 46) = 1.83, *p* = .18. Concerning the CS+, no difference was found between lower and higher scorers (β = -.06, *p* = .35), whereas for the CS-, participants with higher NA reported more pain-related fear compared to those with lower NA (β = .12, *p* = .04).

### Self-reported Behavioural Avoidance Tendency (BAT)

**Fig 3:**
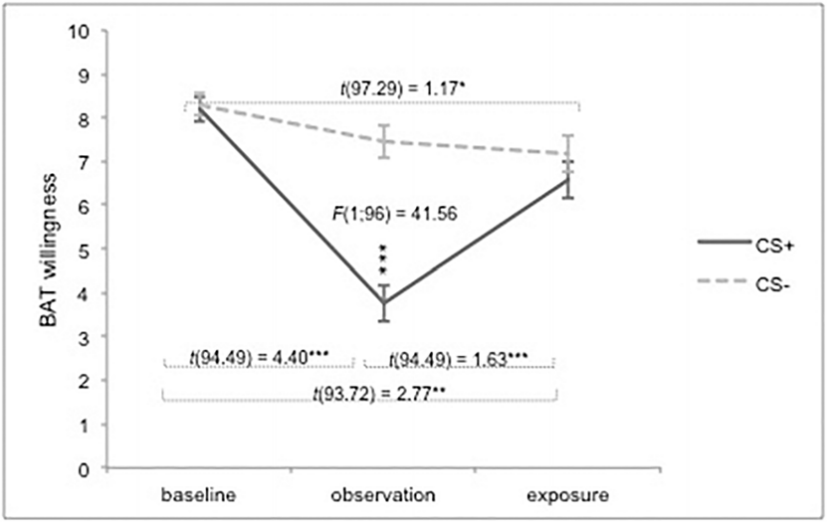
Self-reported behavioural avoidance tendencies in Experiment 1. Willingness to touch the metal bars. Error bars represent standard errors. BAT = Behavioural Avoidance Tendencies; *p < .05; **p < .01; ***p < .001.

As can be seen in Fig. 3, main effects of stimulus type and time, as well as a significant interaction between these two variables were found on self-reported **willingness to touch the bars**, *F*(1;239.84) = 28.72, *p* < .001; *F*(2;241.08) = 32.94, *p* < .001; *F*(2;239.84) = 17.39, *p* < .001, respectively. For the CS+, willingness to touch the bar was significantly lower during the observation phase, compared to the baseline, *t*(94.49) = 4.40, *p* < .001, *r* = .77, and exposure phase, *t*(94.49) = 1.63, *p* < .001, *r* = .49. During exposure, participants were less willing to touch the CS+ bar compared to the baseline phase, *t*(93.72) = 2.77, *p* < .01, *r* = .32. For the CS-, no difference was found between baseline and observation, *t*(97.29) = 0.86, *p* = .13, or between the observation and the exposure phase, *t*(96.56) = 0.31, *p* = 1.00. However, willingness to touch the CS- was significantly lower after exposure compared to the baseline phase, *t*(97.29) = 1.17, *p* = .02, *r* = .26. After watching the observation video, a differential effect was found between the two stimulus types, *F*(1;96) = 41.56, *p* < .001, *r* = .52, with participants being more willing to touch the CS- bar compared to the CS+ bar. No differences between CS+ and CS- were observed during baseline, *F*(1;49) = 0.20, *p* = .65, or during exposure, *F*(1;48) = 2.33, *p* = .13.

**Perspective taking** (PT) was found to moderate the relationship between stimulus type and willingness scores, *F*(4;239.86) = 2.75, *p* = .03, *r* = .20. When conducting separate analyses for acquisition and exposure, we found that PT plays a moderating role on behavioural avoidance tendencies during acquisition (baseline x observation), *F*(1;143.36) = 7.91; *p* < .01, *r* = .28, but not during exposure, *F*(1;48) = 0.17; *p* = .68. Participants with lower PT were more willing to touch the CS- bar relative to the CS+ bar, *F*(1; 46) = 19.39, *p* < .001, whereas for participants with higher PT scores no differential effect was found, *F*(1; 46) = 0.68, *p* = .41. However, regression analyses for each stimulus type separately during acquisition did not reveal any significant effects (CS+: β = .14, *p* = .19; CS-: β = -.17, *p* = .10).

### The Approach Avoidance Task (AAT)

Error rates (inaccurate response direction) were 2% in the baseline phase and 1% in the observation and exposure phase. Corresponding reaction times were excluded from further analyses. No main effects of stimulus type and time, nor an interaction between stimulus type and time was found on the compatibility scores, *F*(1;234.92) = 0.94, *p* = .33; *F*(2;237.36) = 1.16, *p* = .32; *F*(2;234.92) = 1.31, *p* = .27, respectively. None of the observers’ characteristics moderated the relationship between stimulus type and the compatibility scores (all *p* > .05).

#### Skin Conductance Responses (SCR)

*SCR during picture presentation*. A main effect of time was found, *F*(5;528) = 4.61, *p* < .001, with SCR decreasing throughout the exposure phase. No main effect of stimulus type, nor an interaction between stimulus type and time was found regarding SCR to the pictures, *F*(1;528) = 1.35, *p* = .25; *F*(5;528) = 0.40, *p* = .85. Hence, no difference in physiological responding between the reactions to both pictures was observed throughout the exposure phase. None of the observers’ characteristics moderated SCR to the pictures (all *p* > .05).

*SCR during bar exposure*. No effect of stimulus type was found during the presentations of the metal bars, *F*(1;528) = 2.08, *p* = .15. A main effect of time was found, *F*(5;528) = 19.93, *p* < .001, with SCR decreasing during the exposure phase. No interaction between stimulus type and time was found with respect to SCR, *F*(5;528) = 0.31, *p* = .91. Hence, no difference in physiological responding between the two bars was observed throughout the exposure phase, and observers’ characteristics did not influence SCR (all *p* > .05).

## Conclusion

In line with our hypotheses (First aim), participants reported more pain-related fear after watching the observation video with regard to the metal bar that was associated with painful expressions of the video models. Additionally, they expected contact with this metal bar to be more painful in comparison to the coloured bar that was paired with the neutral facial expressions in the video. In contrast to our expectations, these changes in pain-related beliefs did not result in avoidance behaviour with respect to the CS+, although participants reported significantly less willingness to touch the CS+ bar compared to the CS- bar after watching the video clip. No differences in pain-related beliefs between the two bars were found during repeated exposure to the feared stimuli (Second aim). Nor were any differential effects found on psychophysiological responses throughout the exposure phase. Regarding the third aim, perspective taking (PT) moderated the acquisition of pain-related beliefs in the current experiment. Participants with lower PT scores reported significantly more fear and expected more pain intensity with regard to the aversively conditioned stimulus (CS+) in comparison to the neutrally conditioned stimulus (CS-), whereas participants with higher PT did not show a differential effect.

Previous research suggested that being touched by cold metal bars with a temperature of -25°C evokes an ambiguous sensation [20]. In an ambiguous situation, participants were expected to be inclined to rely on information obtained from the environment [53,54], in this case the models’ painful facial expressions in the video clip, to disambiguate the situation in order to interpret their own sensations. Hence, it was hypothesised that watching the video would result in a difference in responding concerning pain-related fear, avoidance behaviour, and psychophysiology between the two metal bars during exposure. A possible explanation for the absence of such a difference in responding in the current experiment could be that the stimuli were considered too aversive, resulting in a ceiling effect, with participants not being able to distinguish sensations regarding both stimuli. This could also explain why self-reported avoidance tendencies after exposure were higher compared to the baseline phase for *both* CS+ and CS- bars. To exclude the possibility that the absence of different responding was merely due to the stimuli being too aversive, a follow-up experiment (Experiment 2) was conducted replicating Experiment 1, but using a higher temperature of the bars.

## Experiment 2: Method

### Participants

Participants were healthy female undergraduate students (N = 43), who received either a course credit or eight Euros for their participation in the study. Exclusion criteria were chronic pain and colour blindness. All participants were of Western European descent, with a mean age of 20.16 years (SD = 1.65; range 18–25). They were told that the study investigated responses to stimuli of different temperatures.

### Ethics statement

Ethical approval for Experiment 2 was obtained from the Ethics Committee of the Faculty of Psychology and Educational Sciences of the University of Leuven, Belgium (Reg. nr. S52888). Participants signed the informed consent form.

### Materials

Materials were the same as used in the first experiment, except for the coloured metal bars, which were cooled down in a refrigerator to approximately +8°C (instead of -25°C) in order to produce a less aversive and more ambiguous sensation.

## Experiment 2: Results

### Sample Characteristics

Descriptive statistics, internal consistency, and Pearson inter-correlations for the different questionnaires and subscales in experiment 2 are summarised in Table 1. Mean scores were comparable to what has been reported in previous research [38,43,45,48].

### Contingency Awareness

All participants were aware of the contingency between the colour of the metal bars and the facial expressions of the video models (painful versus neutral). When dividing the pictures of the video models into two piles, 88.4% of the participants correctly categorised all six CS+ pictures as painful, and 11.6% erroneously categorised one CS+ picture as neutral.

### Self-reported Pain-related Fear and Pain Intensity

#### Baseline and Observation Phase

**Fig 4:**
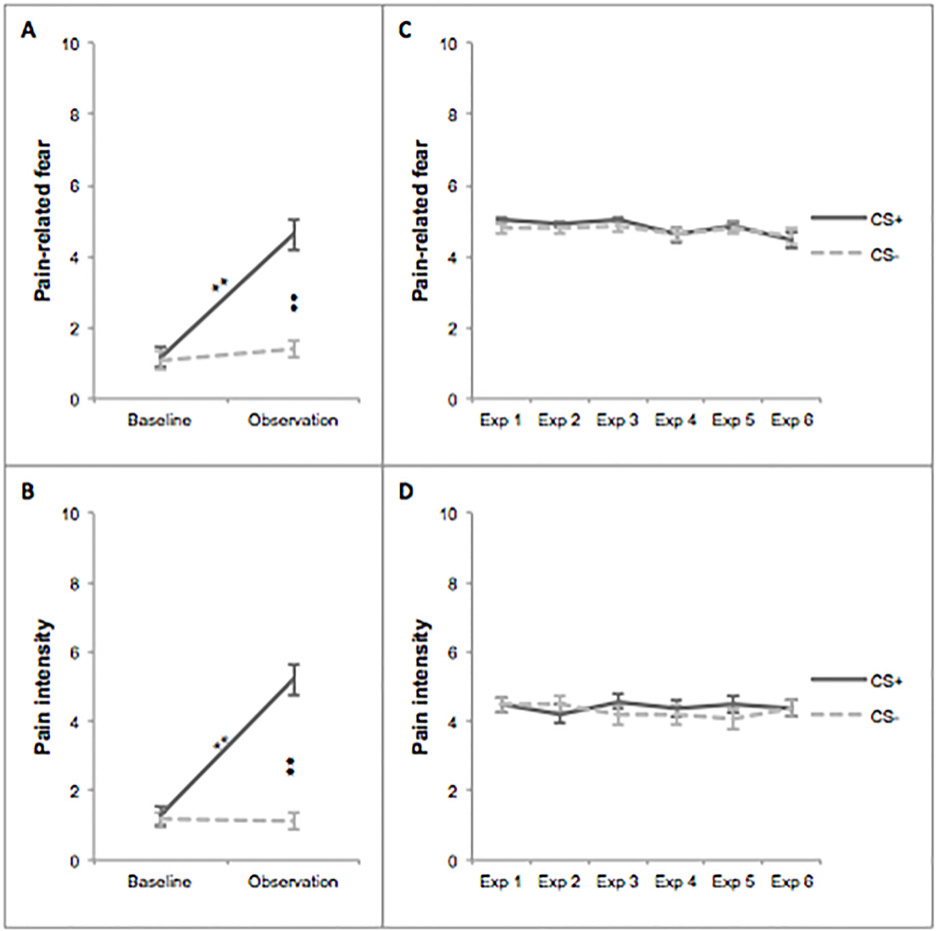
Self-reports: Pain-related fear and pain intensity in Experiment 2. Error bars represent standard errors. Exp = Exposure; * *p* < .05; ** *p* < .001.

Results were very similar to the results of Experiment 1. With regard to **pain-related fear**, which is illustrated in Fig. 4A, main effects of stimulus type and time were found, *F*(1;129) = 42.90, *p* < .001, effect size *r* = .59; *F*(1;129) = 58.87, *p* < .001, *r* = .08, respectively. Additionally, a significant interaction was found between stimulus type and time, *F*(1;129) = 40.49, *p* < .001, *r* = .46. No difference in fear between both stimulus types was reported during the baseline, *F*(1; 43) = 0.04, *p* = .84. During the observation phase, however, more fear was reported concerning the CS+ bar compared to the CS- bar, *F*(1; 43) = 72.38, *p* < .001, *r* = .75. This difference was due to an increase in fear regarding the CS+, *F*(1;43) = 63.43, *p* < .001, *r* = .73, as no difference was found between the two phases with respect to the CS-, *F*(1;43) = 1.51, *p* = .23.

Furthermore, Fig. 4B presents main effects of stimulus type and time on **pain intensity expectancies**, *F*(1;129) = 62.56, *p* < .001, *r* = .69; *F*(1;129) = 53.21, *p* < .001, *r* = .01. In addition, a significant interaction was found between stimulus type and time, *F*(1;129) = 55.81, *p* < .001, *r* = .55. During the baseline, participants reported no difference in expected pain intensity between both stimulus types, *F*(1; 43) = 0.28, *p* = .60, whereas during the observation phase, contact with the CS+ bar was expected to be more painful than the CS- bar, *F*(1;43) = 81.71, *p* < .001, *r* = .81. This differential effect was caused by an increase in pain intensity expectancy regarding the CS+, *F*(1;43) = 84.09, *p* < .001, *r* = .81, as no difference between baseline and observation was found for the CS- bar, *F*(1;43) = 0.02, *p* = .88.

#### Influence of Observers’ Characteristics during the Baseline and Observation Phase

Only statistically significant effects are reported and explained. **Fantasy** (FS), which is the tendency to identify strongly with the feelings and actions of others, moderated the relationship between stimulus type and expected pain intensity, *F*(1;129) = 5.67, *p* = .02, *r* = .25. Participants expected more pain intensity regarding the CS+ compared to the CS-, and these differential effects were even stronger for participants scoring higher on FS, *F*(1; 46) = 52.87, *p* < .001, relative to participants with lower FS, *F*(1; 46) = 15.73, *p* < .001. Regression analyses for both stimulus types separately did not reveal any statistically significant results (CS+: β = .16, *p* = .14; CS-: β = -.08, *p* = .47).

#### Exposure Phase

The results of the exposure phase were comparable to the results obtained in Experiment 1, which is illustrated in Fig. 4. As shown in Fig. 4C, regarding **pain-related fear**, no main effect of stimulus type, nor an interaction between stimulus type and time was found, *F*(1;516) = 0.20, *p* = .66; *F*(5;472) = 0.37, *p* = .87, respectively. However, in Experiment 2, a main effect of time was found regarding pain-related fear, *F*(5;472) = 2.73, *p* = .02, with fear decreasing throughout the exposure phase. As illustrated in Fig. 4D, no main effects of stimulus type, *F*(1;516) = 0.01, *p* = .91, and time, *F*(5;516) = 0.18, *p* = .97, nor an interaction between these two variables, *F*(5;516) = 0.56, *p* = .73, was found for **pain intensity** during the exposure phase. To summarise, no difference between both stimuli was found throughout the exposure trials. Baseline expectancy ratings did not influence the level of experienced pain-related fear, *F*(1;64.96) = 0.67, *p* = .42, and pain intensity, *F*(1;516) = 2.04, *p* = .15, during the exposure phase.

#### Influence of Observers’ Characteristics during the Exposure Phase

No statistically significant moderation effects were found during exposure.

### Self-reported Behavioural Avoidance Tendency

**Fig 5:**
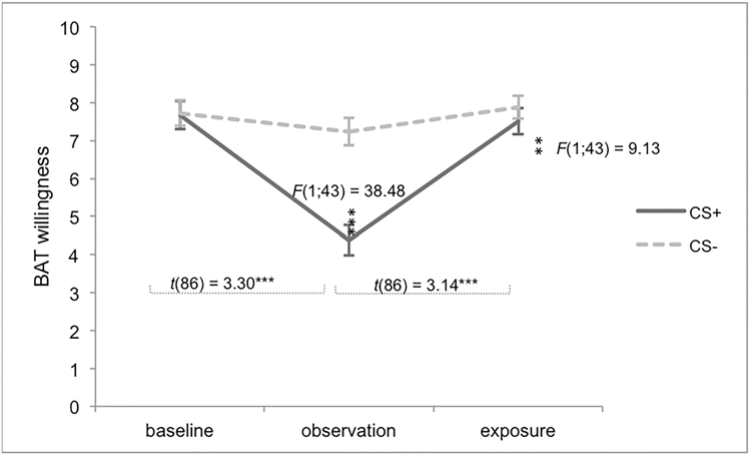
Self-reported behavioural avoidance tendencies in Experiment 2. Willingness to touch the metal bars. Error bars represent standard errors. BAT = Behavioural Avoidance Tendencies; *p < .05; **p < .01; ***p < .001.

Results of the self-reports concerning behavioural avoidance tendencies were provided in Fig. 5, and comparable to the results of Experiment 1. In contrast to the first experiment, willingness to touch the CS+ bar after exposure did not differ from baseline level, *t*(86) = 0.16, *p* = 1.00, although willingness to touch the CS+ bar was still significantly lower as compared to the CS- bar, *F*(1;43) = 9.13, *p* = .004, *r* = .36. No moderating effects were observed.

### The Approach Avoidance Task (AAT)

Error rates (inaccurate response direction) were 1% in all three phases, and corresponding responses were excluded from further analyses. No main effect of stimulus type or time, nor an interaction between these two variables was found on the compatibility scores, *F*(1;215) = 1.69, *p* = .20; *F*(2;215) = 1.03, *p* = .36; *F*(2;215) = 1.00, *p* = .37, respectively. This means that overall, no differences between stimulus types were found over time.

**Trait fear of pain** (FPQ) moderated the relationship between stimulus type and compatibility scores, *F*(4;215) = 2.62, *p* = .04, *r* = .23. When conducting separate analyses for acquisition and exposure, trait fear of pain was found to play a moderating role on AAT compatibility scores during the acquisition phase (baseline x observation), *F*(1;129) = 5.51; *p* = .02, *r* = .27, but not during the exposure phase, *F*(1;43) = 0.00, *p* = 1.00. Participants with higher trait fear of pain showed more approach tendencies regarding the CS+ relative to the CS- pictures during acquisition, *F*(1; 45) = 6.59, *p* = .01, while for participants scoring lower on the FPQ, no differential effects were found, *F*(1; 45) = 0.92, *p* = .34. Regression analyses for each stimulus type separately during acquisition revealed that regarding the CS+ metal bar, participants scoring lower on the FPQ showed relatively more avoidance behaviour, whereas participants scoring higher on the FPQ displayed relatively more approach tendencies (CS+: β = .32, *p* < .01). With regard to the CS- bar, no differences were found between lower and higher scorers on the FPQ (CS-: β = .07, *p* = .52).

#### Skin Conductance Response (SCR)

*SCR during picture presentation*. A main effect of time was found, *F*(5;473) = 2.31, *p* = .04, with SCR decreasing over time. No main effect of stimulus type, nor an interaction between stimulus type and time was found with respect to SCR, *F*(1;473) = 0.15, *p* = .70; *F*(5;473) = 0.60, *p* = .70. Although physiological reactions diminished, no difference in psychophysiological responses was found between the CS+ and CS- bar.

Statistically significant moderation effects are notified. **Fantasy** (FS), **personal distress** (PD), and **negative affectivity** (NA) had a moderating influence on SCR to the pictures of the bars, *F*(1;473) = 12.37, *p* < .001, *r* = .40; *F*(1;473) = 6.38, *p* = .01, *r* = .30; *F*(1;473) = 7.18, *p* = .01, *r* = .32, respectively. Participants with lower FS showed stronger physiological responses when watching the CS+ pictures compared to the CS- pictures in anticipation of exposure to the bars, *F*(1; 46) = 4.25, *p* = .046, while no differential effects were found for participants with higher FS, *F*(1; 46) = 2.77, *p* = .10. For PD and NA, no differential effects were found for participants scoring lower, *F*(1; 46) = 2.29, *p* = .14; *F*(1; 46) = 2.58, *p* = .12, or higher on these personality traits, *F*(1; 46) = 1.28, *p* = .27; *F*(1; 46) = 1.49, *p* = .23. Regarding the CS+ bar, participants scoring lower on FS, or NA showed stronger physiological reactions compared to participants scoring higher on these questionnaires (β = -.18, *p* = .01; β = -.21, *p* = .001, respectively). With respect to PD, no difference was found between lower and higher scorers concerning the CS+ (β = .09, *p* = .15). For the CS- bar, participants scoring higher on PD displayed stronger psychophysiological reactivity than participants scoring lower (β = .25, *p* < .001), while for FS and NA, no difference in physiological responding was found (β = .06, *p* = .35; β = -.02, *p* = .73).

*SCR during bar exposure*. A main effect of stimulus type was found during exposure to the metal bars, *F*(1;473) = 4.35, *p* = .04, *r* = .02. As expected, participants showed more SCR with regard to the CS+ compared to the CS- bar. A main effect of time was found, *F*(5;473) = 12.14, *p* < .001, with SCR decreasing throughout the exposure phase. No interaction between stimulus type and time was found with respect to SCR, *F*(5;473) = 1.07, *p* = .38, indicating that no difference in the reduction of the physiological responses was found between the CS+ and CS- bar throughout the exposure phase. None of the investigated observers’ characteristics was found to have a moderating influence on SCR to the presentation of the bars (all *p* > .05).

## Discussion

The main objective of the current study was to examine observational acquisition of pain-related fear (First aim) and the subsequent extinction through repeated first-hand exposure to the feared stimuli (Second aim), focussing on (1) self-reports, (2) psychophysiological responses, and (3) behavioural tendencies. Moreover, the study investigated which individual characteristics predict observational pain-related fear acquisition (Third aim). A differential fear conditioning procedure was used, showing video models displaying either painful (CS+ colour) or neutral (CS- colour) facial expressions when exposed to one of two coloured metal bars (observation phase). Afterwards, both metal bars with equal temperatures (Experiment 1: -25°C; Experiment 2: +8°C) were repeatedly presented to participants’ necks (exposure phase).

Regarding the first aim, in the current study, evidence was found for indirect acquisition of pain-related fear beliefs, corroborating earlier findings [19]. After watching the observation video, participants reported more pain-related fear regarding the coloured bar that was previously associated with models’ painful expressions. They also expected contact with this bar to be more painful compared to the bar that was paired with the models’ neutral expressions, and were less willing to touch it.

In contrast to earlier findings in a non-pain context [55], changes in pain-related beliefs did not result in CS+ avoidance, which was measured implicitly using an approach-avoidance task. A possible explanation could be the (lack of) personal relevance of the picture stimuli. Fear conditioning takes place at different levels: The automatic associative level (emotional), and the non-automatic cognitive contingency level [56,57]. Fear-relevant stimuli address both levels independently, while the use of fear-irrelevant stimuli leads to activation of only the cognitive level. The laboratory setting might not have been threatening for healthy participants, whereas for pain patients, impending pain is more salient, resulting more easily in behavioural changes, especially when using implicit (non-cognitive) measures. In previous fear research [58,59], avoidance behaviour was measured using a behavioural approach-avoidance paradigm, in which participants gradually approached the feared stimulus. However, in the current study, touching the bars after watching the video would interfere with experiences during exposure. Comparing latency time before contact with the feared and non-feared stimulus [19] was not an option either because participants could not control the exact moment of exposure.

Concerning the second aim, no differences between both bars were observed regarding pain-related fear and pain intensity during repeated exposure. Nor did the study find a reduction in these measures throughout this phase, although a small overall decrease in pain-related fear was found in the second experiment. The absence of a difference might be due to generalisation of the fear to the CS-, as both coloured bars share several features [60]. During acquisition, expectations were measured, whereas during exposure, actual experiences were investigated, which complicates comparison of the different phases. Nevertheless, it seems that not the negative appraisals regarding the CS+ have diminished after exposure, but rather the aversive beliefs concerning the CS- were enhanced. Results regarding the self-reported avoidance tendencies after exposure were not unequivocal. In the first experiment, no difference between the two stimuli was found, although willingness to touch both bars was lower compared to the start of the experiment. These findings might indicate that a temperature of -25°C may have been too aversive, resulting in avoidance of both bars. In the second experiment, presumably using a more ambiguous temperature, willingness to touch the CS- bar was higher compared to the CS+ bar, and did not differ from baseline. One could argue that these results provide evidence for the persistence of differential fear until the end of the experiment.

In contrast to earlier findings regarding fear in general [61,62], no differences in SCR in anticipation of direct contact with the bars were observed in either experiment. Only in the second experiment, evidence was found for differential psychophysiological responding during contact with both metal bars, and persisted throughout exposure. Higher values for both metal bars in Experiment 1 might suggest that the temperature was too aversive. It is, however, difficult to compare these physiological findings with previous research, as in other paradigms no real shocks [18] or enriched CO_2_ air [62] were administered during the test phase.

Examining putative moderators is essential in the identification of individuals at risk of developing pain problems (Third aim). In this study, none of the investigated moderators had a consistent influence across both experiments. Some trends can be observed, which should, however, be interpreted cautiously. In the first experiment, participants with lower tendency to adopt another’s viewpoint (perspective taking; PT) reported significantly more fear, and expected more pain intensity regarding contact with the CS+ compared to the CS-, whereas for participants with higher PT no differential effects were found. Similar results were found for self-reported behavioural avoidance tendencies. A possible explanation, in line with the findings of Mailhot and colleagues [63], might be that individuals with higher PT focus on the emotional reaction of the model in pain, possibly neglecting the information which might be useful for their own experience. Prior studies have shown that viewing pain in others facilitates pain-related fear responses [63]. However, observers with higher dispositional empathy displayed a reduction of the observational facilitation of perceptual pain responses (pain intensity, pain unpleasantness), although the NFR (nociceptive flexion reflex) was not affected by prior observational learning [63,64]. This was explained by the distinction between low level empathic processes (e.g., emotional contagion), which occur automatically during the first stage of a pain experience, and high level empathic processes (e.g., perspective taking and mentalising), which are driven by higher cognitions and may lead to suppression of automatic defensive responses during the second stage of a pain experience [65]. Such down-regulation of self-protective responses enables highly empathic individuals to remove attention from their own discomfort, and to display prosocial behaviour. Hence, a potential explanation for the differential effects regarding PT on fear beliefs in low but not high PT might be due to an attentional bias to others’ emotional responses in individuals with high PT. This may have distracted them from the other stimuli presented in the video, which resulted in disturbed discrimination learning concerning the CS+ and CS- metal bar. In the second experiment, participants with higher fantasy (FS), who strongly identify with feelings and actions of others, discriminated better between CS+ and CS- in terms of their expected pain intensity relative to participants with lower FS. Although the current findings suggest that dispositional empathy may play a role in the observational acquisition of pain-related beliefs, none of the investigated empathy subscales had a consistent influence across both experiments, necessitating caution in interpretation and further investigation.

In contrast to our expectations, participants scoring higher on trait fear of pain in Experiment 2 displayed relatively more approach tendencies concerning the CS+ bar after watching the video clip than those with lower scores. This rather contradictory result might be related to the scant threat value of the picture stimuli. The prospect of contact with the CS+ metal bar is considered threatening by the participants. Although a picture of this threatening CS+ bar was presented during the AAT, there was no real need to avoid actual danger or contact with the feared stimulus at this stage, as it was just a picture on a computer screen. For participants with lower trait fear of pain, no difference was found between compatibility scores for both stimulus types. For participants with higher trait fear of pain, more approach tendency concerning the CS+ picture was found compared to the CS- picture. There might be at least two explanations. First, the CS+ picture might not have been threatening enough, but still may cause some discomfort in the high fearful observer. Hence, the use of a CS+ metal bar picture in the AAT might not have had the same threat value as the real CS+ metal bar. Second, faster movements when a ‘pull’-movement was required resulted in quicker disappearance of this putative discomfort caused by watching the approaching stimulus in the picture in individuals with higher trait fear of pain. In that sense, a pull movement might also have functioned as an active avoidance response. It was, however, difficult to apply a ‘real’ behavioural approach task (e.g. [66]) in this experiment, as actual contact with the metal bars during the different phases of the experiment would have interfered with the flow of the experiment. Moreover, individuals with higher fear of pain are likely to selectively attend to potentially threatening stimuli in their environment [67]. The ability to orient away from pain-related stimuli may be under conscious control in low fearful people, whereas such a mechanism does seem absent in those high in the fear of pain [68,69]. Another possibility might be that the optical zooming illusion did not have the intended effect. On the other hand, participants with higher perspective taking, personal distress, and negative affectivity (NA) showed stronger fearful responses to CS-, suggesting that they may be deficient in safety learning. This corroborates earlier findings showing that NA is associated with poorer discrimination between safe and unsafe stimuli, more readily considering safe stimuli as potentially unsafe [70,71]. The findings regarding empathic tendencies (PT, PD) are in line with Valeriani et al. [26], who suggest that disturbed discrimination learning might be due to an attentional bias to others’ emotional responses in highly empathic individuals.

These results may yield some implications for clinical pain management. Since pain-related fear can be more disabling than the pain itself [41], many pain patients may benefit from treatment targeting pain-related fear. It might also be appropriate to implement knowledge concerning observational learning in acute pain situations, because early prevention interventions concerning pain-related fear may avert transition from acute to chronic pain. For instance, health care professionals should be aware of their attitudes regarding pain and pain-related fear [72,73], as patients might take over these attitudes through observation and verbal instructions. Additionally, meeting recovered pain patients suffering from similar injuries and observing them performing daily back-stressing movements and activities might reduce pain-related fear acquisition. Furthermore, family members of pain patients can be involved in psycho-educational treatment sessions explaining observational learning processes and possible maintaining factors concerning pain, since these individuals often observe their family member in pain, which may increase their own vulnerability for pain, avoidance and disability later in life [74]. No strong evidence was found in this study regarding the implications for exposure therapy, although some findings might be promising. Although no differential effects in fear beliefs were observed during the exposure phase, in the second experiment, an overall decrease of pain-related fear was observed throughout the exposure trials. In addition, willingness to touch the CS+ metal bar significantly increased during the exposure phase in both experiments.

There are a few limitations to this study, which need to be considered. First, participants were healthy young females, restricting external validity. Previous research has shown that gender differences exist in pain experience, possibly due to different operating pain mechanisms [75]. Moreover, observers might learn more easily from same-gendered models or from ‘in-group’ members [76]. Both the models and the participants in our study were young females. The models were told to be students that participated earlier in the same study, hence belonging to the same in-group. The current study has only focused on females, because women are more prone to develop chronic pain, and are also known to report having more pain models, who are mostly female [77]. Future research is needed to examine whether our findings generalise to male samples and individuals suffering from acute or chronic pain. Second, skin conductance was used as a psychophysiological measure. Startle response (EMG) might be a better measure to use in future experiments because it is more specifically related to fear, whereas skin conductance is a measure for general arousal [78]. In addition, it has been used successfully in experimental fear of pain studies [79]. Third, a different behavioural measure could be applied in future research, as there might be some difficulties concerning insufficient threat value of the picture stimuli used in the AAT.

Despite these limitations, the findings of this study may enhance our understanding of observational pain-related fear acquisition and subsequent first-hand exposure, providing leads for pain prevention and management strategies.
